# Perlecan Maintains Microvessel Integrity *In Vivo* and Modulates Their Formation *In Vitro*

**DOI:** 10.1371/journal.pone.0053715

**Published:** 2013-01-08

**Authors:** Erika Gustafsson, Maylin Almonte-Becerril, Wilhelm Bloch, Mercedes Costell

**Affiliations:** 1 Department of Experimental Pathology, Lund University, Lund, Sweden; 2 Department of Biochemistry and Molecular Biology, University of Valencia, Valencia, Spain; 3 Departamento de Infectómica y Patogénesis Molecular, Centro de Investigación y de Estudios Avanzados del Instituto Politécnico Nacional, México Distrito Federal, México; 4 Department of Molecular and Cellular Sport Medicine, Cologne, Germany; University of Bergen, Norway

## Abstract

Perlecan is a heparan sulfate proteoglycan assembled into the vascular basement membranes (BMs) during vasculogenesis. In the present study we have investigated vessel formation in mice, teratomas and embryoid bodies (EBs) in the absence of perlecan. We found that perlecan was dispensable for blood vessel formation and maturation until embryonic day (E) 12.5. At later stages of development 40% of mutant embryos showed dilated microvessels in brain and skin, which ruptured and led to severe bleedings. Surprisingly, teratomas derived from perlecan-null ES cells showed efficient contribution of perlecan-deficient endothelial cells to an apparently normal tumor vasculature. However, in perlecan-deficient EBs the area occupied by an endothelial network and the number of vessel branches were significantly diminished. Addition of FGF-2 but not VEGF_165_ rescued the in vitro deficiency of the mutant ES cells. Furthermore, in the absence of perlecan in the EB matrix lower levels of FGFs are bound, stored and available for cell surface presentation. Altogether these findings suggest that perlecan supports the maintenance of brain and skin subendothelial BMs and promotes vasculo- and angiogenesis by modulating FGF-2 function.

## Introduction

The formation of an elaborate vascular network is critical for development, tissue repair and tumor growth. During development, the vascular system forms by two different processes termed vasculo- and angiogenesis. During vasculogenesis mesenchymal progenitor cells differentiate into endothelial cells and establish a primitive vascular plexus. Angiogenesis occurs when endothelial cells start to proliferate and to sprout from preexisting vessels and thereby forming new vessels. The newly formed endothelial tubes finally mature by assembling a basement membrane (BM) and by recruiting smooth muscle cells or pericytes [1]. The formation and maturation of blood vessels are tightly controlled by a finely tuned balance of pro- and anti-angiogenic actions executed by growth factors, cell adhesion molecules and components of the extracellular matrix ECM.

Perlecan is a major a heparan sulfate (HS) proteoglycan associated with blood vessels. Its highest expression in the mammalian embryo coincides with the development of blood vessels (E10.5 in the mouse) and the heart. Later in development it is expressed in most visceral organs, skeletal muscle and cartilage [2], [3]. Perlecan is deposited in vascular BMs, in the mesenchyme of several developing organs, and in the stroma of wounds and tumors. The targeted deletion of the perlecan gene in mouse revealed a critical role for the development of cartilage [4], [5], heart [6] and brain [7]. Perlecan deficiency is embryonic lethal. Mutant embryos die between E10.5 and around birth. Laminin, collagen IV, nidogen and perlecan are major components of BMs. Although perlecan is not essential for BM assembly it is important to maintain BM integrity in several tissues. For example, perlecan-null embryos display disrupted BMs in the developing heart [8] and around the expanding telencephalic vesicles leading to neuronal ectopias or exencephaly [7]. Other critical functions of the protein include neuroblast proliferation [7], endochondral ossification [5], acetylcholinesterase clustering at the neuromuscular junctions [9] or proliferation of mesenchymal cells in the endocardial ridges that form the heart outflow tract [6]. In humans, mutations in the perlecan gene cause *Schwartz-Jampel* syndrome, a disorder consisting of chondrodysplasia and myotonia [10], [11].

One of the most intriguing functions of perlecan is its involvement in blood vessel formation. The mRNA levels of perlecan are high in endothelial cells of the developing mouse embryo [2], and further increase after recruitment of pericytes to the endothelial tubes. Both cell types contribute to the secretion and assembling of the vascular BM [1]. Perlecan together with other proteins, such as fibronectin, nidogen and laminin, are progressively remodeled by integrins to finally ensheath the mature vessel [12], [13].

Perlecan can exert promoting as well as inhibiting roles during angiogenesis (reviewed in [14], [15]). Suppression of perlecan expression in a colon cancer cell line or in metastatic prostate cancer cells [16] for example, leads to diminished tumor angiogenesis and tumor growth. In fibrosarcoma, however, the opposite effect was observed [17]. Perlecan can bind pro-angiogenic factors including growth factors such as fibroblast growth factors (FGFs), vascular endothelial growth factor (VEGF), platelet-derived growth factor-B (PDGF-B), transforming growth factor-β (TGF-β), β1 and β3 integrins and fibronectin. Apart from binding growth factors, perlecan can also present angiogenic growth factors to their receptors [18] or prevent growth factor receptor signaling through binding them. The latter was shown for VEGF-A which cannot bind VEGFR2 in the presence of perlecan [19]. Furthermore, perlecan also serves as a slow release reservoir of growth factors and protects them from proteolysis [20]. It has also been shown that proteolytic cleavage products of BM constituents can serve as potent inhibitors of tumor angiogenesis. They can be derived from collagens type IV and XVIII, laminins and also perlecan (reviewed in [21]). The C-terminal domain V of perlecan is called endorepellin and was shown to inhibit angiogenesis [22], [23]. Apart from these anti-angiogenic properties of fragments from perlecan, perlecan may also interact with other HS-binding regulators of angiogenesis such as thrombospondin [24], endostatin [25], NK4 [26] and platelet factor-4 [27] and thus indirectly contribute to angiogenesis. Finally, a key role of perlecan is also to maintain BMs, which constitute a structural and functional microenvironment for vascular cells.

In spite of the extraordinary amount of information about the role of perlecan in angiogenesis in vitro, it is still not clear whether perlecan is also involved in the regulation of de novo vasculogenesis and angiogenesis. For example, morpholino-mediated knockdown of perlecan in zebrafish produces anomalous development of the intersegmental and dorsal longitudinal vessels [28]. Mice engineered to express a HS-deficient perlecan are born normally and have a normal blood vessel development [29], although they suffer from lens degeneration and aberrant wound healing, tumor growth and impaired corneal angiogenesis [30].

In the present paper we tested the role of perlecan in vessel formation by disrupting the perlecan gene in mice, teratomas and embryoid bodies (EBs). We demonstrate no rate limiting function for perlecan in vasculo- and angiogenesis but a critical role in maintaining microvessel integrity in vivo. Furthermore, we show that perlecan does not modulate teratoma angiogenesis, but controls the vascular network in EBs through its ability to store and/or present FGF-2.

## Materials and Methods

### Ethics Statement

The mice used for this study were kept in the animal house of the Max Planck Institute of Biochemistry. The analysis of perlecan-null mice was carried out in strict accordance with all German (e.g. German Animal Welfare Act) and EU (e.g. Directive 86/609/EEC) applicable laws and regulations concerning care and use of laboratory animals. The Max Planck Institute of Biochemistry has a license for breeding and housing laboratory animals (No. 5.1-568 - rural districts office). All animals used were bred for scientific purposes. The Max Planck Institute of Biochemistry is registered at NIH and has a PHS Approved Animal Welfare Assurance from the Office of Laboratory Animal Welfare: #A5132-01 (see: http://grants.nih.gov/grants/olaw/assurance/500index.htm?Country=GM#GridTop).

### Mice, ES Cells and Antibodies

Perlecan-null mice and ES cells were generated by gene targeting and have been described previously [5]. Briefly, the targeting construct was electroporated into ES derived from 129/Sv mice. The ES cell clones were injected into blastocysts from C57BL6 females to generate germline chimeras.

The following primary antibodies were used for immunohistochemistry: rabbit anti-laminin-1; rabbit anti-perlecan antibodies against domain V; rabbit anti-nidogen-1; rabbit anti-collagen IV; rabbit anti-collagen XVIII against endostatin (obtained from Dr. Rupert Timpl); rabbit anti-desmin (Sigma-Aldrich); rabbit anti-NG2 (Millipore); rabbit anti PDGFR (Cell Signaling); rat anti-flk-1 (obtained from Dr. Urban Deutsch, Bad Nauheim, Germany); rabbit anti-fibronectin (Chemicon); rat anti-PECAM-1 (Pharmingen) and mouse anti-smooth muscle cell a-actin (Sigma-Aldrich). The following secondary antibodies were used: biotinylated goat anti-rabbit IgG, biotinylated goat anti-rat IgG, biotinylated goat anti-mouse IgG, biotinylated rabbit anti-goat IgG (all obtained from Vector Laboratories Inc.); FITC-conjugated goat anti-rabbit, Cy3-conjugated goat anti-rat (both obtained from Jackson Immunoresearch Laboratories Inc.).

To quantify vessel size in the cortical primordium of E15.5 embryos, vessel perimeters from three mutant and wild type embryos were measured in four areas of three coronal sections immunostained for laminin111 using ImageJ (NIH, USA). The results were statistically evaluated using a two-tailed Mann-Whitney Test.

### Generation of Teratomas

For teratoma induction, wild type and perlecan-null ES cells were trypsinized, washed twice in PBS, suspended in PBS at a concentration of 10^8^ cells/ml, and a 100 µl cell suspension (10^7^ cells) was injected subcutaneously on the back of syngeneic 129/Sv male mice. After 28 days, the tumors were surgically removed, weighed and frozen in ice-cold isopentane.

### Generation and Analysis of Embryoid Bodies

For *in vitro* differentiation in EBs, wild type and mutant ES cells were cultured in hanging drops as described elsewhere [31]. Briefly, 600 cells were cultured in 20 µl of DMEM, supplemented with 20% fetal calf serum (FCS), non-essential amino acids and 0.1 mM β-mercaptoethanol, hanging from the lid of a Petri dish for 2 days allowing the formation of cell aggregates and then for 3 days in bacteriological Petri dishes. Subsequently, the aggregates were plated on Tissue Tek chambers pre-coated with 0.1% gelatin and incubated for 5–12 days in 20%, 10%, 5%, 2% or 0.5% FCS-containing medium. Perlecan with or without HS side chains (10 ng/ml, 100 ng/ml and 500 ng/ml) purified from the EHS tumor [2] was added to the EB cultures in rescue experiments. To evaluate the effect of growth factors, EBs were cultured in the presence of VEGF_165_ (1 nM and 3 nM; R&D Systems), human FGF-2 (0.3 nM, 0.6 nM and 1.2 nM; Cell Concepts) and a combination of both VEGF_165_ and FGF-2 (1 nM and 1.2 nM, respectively). The EBs were immunostained as previously described [32]. The number of PECAM-positive vessel branch-points was counted in randomly selected areas of 1.5 mm^2^. The size of the vascularized area was measured on low-magnification images using the Axiovision Software (Zeiss). The resulting data was statistically evaluated using a two-tailed Mann-Whitney Test.

### FGFR1-AP Preparation and in Situ assay for Quantification of FGF-2 Binding to FGFR1 in EBs

An expression construct encoding the extracellular domain of fibroblast growth factor receptor 1 (FGFR1) linked to human placental alkaline phosphatase (AP) was kindly provided by Dr. David Ornitz. This construct has successfully been used to determine binding of FGF-2 and FGFR1 in cells and tissues [33]. The expression vector FGFR1-AP in pcDNA3 (Invitrogen) was linearized and transfected into human embryonic kidney 293 cells (HEK 293 cells; American Type Culture Collection) with LipofectAMINE reagent (Invitrogen) and selection was carried out with 800 µg/ml G418 (Geniticin, Life Technologies, Inc.-BRL). G418-resistant clones were isolated, expanded and assayed for AP activity according to Flanagan and Leder [34]. Conditioned serum-free medium was collected from the highest expressing clone, dialysed in 20 mM triethanolamine (pH 7.5), 50 mM NaCl and applied on a 2 ml bed volume Q-sepharose Fast Flow (Amersham Biosciences) column equilibrated with the same buffer. The FGFR1-AP protein was eluted by a linear gradient from 50 mM to 1 M NaCl over 20 bed volumes at 1 ml/min, and 0.5-ml fractions were collected. Fractions containing FGFR1-AP were identified by AP activity and the protein purity was judged by 7.5% SDS-PAGE and Coomassie R-250 Brilliant Blue staining. The positive fractions were pooled and dialyzed in PBS. FGFR1-AP concentration was determined by BCA protein assay (Pierce, Rockford, IL). For the binding assay, wild type and perlecan mutant EBs were grown until confluency in 96-well plates and fixed with 4% paraformaldehyde in PBS for 20 minutes. After washing in PBS, EBs were incubated with 10 nM-500 nM of recombinant FGF-2 in PBS +0.1% BSA for 1 h at 37°C. After rinsing, FGFR1-AP was added at a concentration of 300 µg/ml for 1 h at 37°C. Unbound receptor was removed by washing and immobilized AP activity was determined by adding an AP substrate mix to the wells as described above. The resulting data were statistically evaluated using a two-tailed Students *t*-test.

### Whole-mount Immunohistochemistry

For whole embryo immunohistochemistry staged embryos were dissected in PBS. Part of the yolk sac was used for genotyping by Southern blot. Yolk sacs and embryos were fixed overnight in Dent's fixative (80% methanol: 20% DMSO). The samples were incubated in 6% H_2_O_2_ in methanol for 1 hr at room temperature to quench endogenous peroxidases. The samples were then rehydrated to PBS with 0.1% Tween-20, incubated in antibody buffer (10% goat serum, 5% BSA in PBS) 2×1 hr, and exposed to PECAM-1 or flk-1 rat monoclonal antibody overnight at 4°C After 5–7 hr washes in 0.1% Tween-20 in Tris-buffered saline, samples were incubated with alkaline phosphatase-conjugated goat-anti rat IgG (Boehringer Mannheim). The detection system was NBT/BCIP (Boehringer Mannheim). Stained embryos and yolk sacs were post-fixed with 10% formaldehyde, examined, and photographed on a dissecting microscope.

### Histological Analysis, Immunohistochemistry and Electron Microscopy

Embryos were either fixed in 4% fresh paraformaldehyde in PBS, pH 7.2, overnight, dehydrated in graded alcohol series and embedded in paraffin (Paraplast X-tra; Sigma) or directly embedded in tissue freezing medium (Tissue Tek, Leitz Industries). Sections were cut at 6–8 µm. Staining with haematoxylin and eosin was performed using standard procedures and immunostainings either with the Vectastain ABC Elite kit (Vector Laboratories) or an immunofluorescence method described previously [7]. Ultrastructural analysis of BMs was performed as described [8].

## Results

### Early Vasculogenesis and Angiogenesis is not Altered in the Absence of Perlecan

To evaluate the role of perlecan in vasculo- and angiogenesis we analyzed 46 perlecan-null embryos and 90 wild-type littermates, ranging from E9.0 to E17.5. Until E12.5 perlecan-deficient embryos displayed a normal appearance and were indistinguishable from wild type controls. To test whether the lack of perlecan affects vasculogenesis and angiogenesis, whole mount immunohistochemical stainings were performed with an antibody recognizing PECAM-1, a marker for endothelial cells. At E9.5 PECAM-1 staining was indistinguishable between normal and perlecan-null embryos (Fig. S1 A–D). Perineural capillary plexus, intersomitic vessels and dorsal aorta were well-developed and contained blood cells. The extra-embryonic vasculature analyzed in the yolk sacs at E10.5–18.5 using anti-PECAM-1 and anti-flk-1 antibodies showed normal expression of these endothelial markers (Fig. S1 E–H and not shown). We quantified the density of the capillary networks by counting the number of vessel branch points per mm^2^ in the dorsal perineural area of E9.5 brain sections and in E10.5 and E14.5 yolk sacs (Fig. S1I). The number, size and shape of vessels were similar between control and mutant. These data suggest that perlecan is not essential for early vasculo- and angiogenesis and remodeling of capillaries into different vessel sizes.

### Perlecan-null Microvessels Dilate and Rupture at Later Developmental Stages

**Figure 1:**
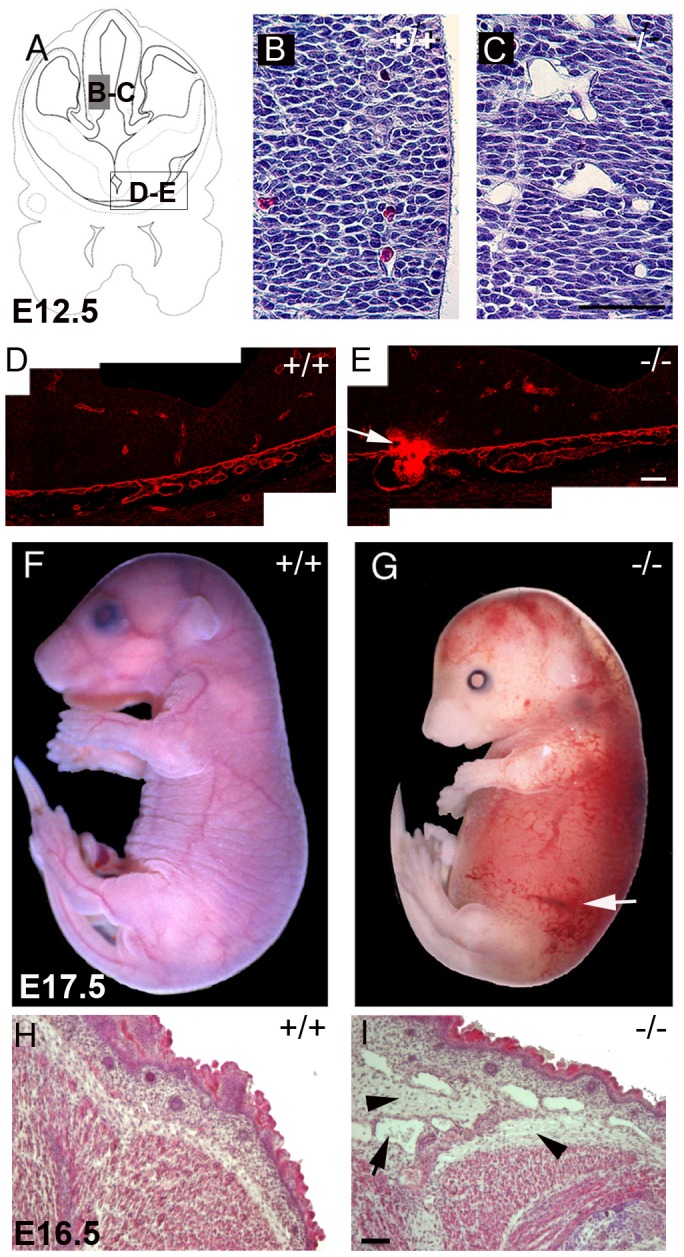
Vascular defects in perlecan-null embryos. (A) Schematic view of the region of the forebrain shown in (B–E). (B–C) Nissl stained coronal sections of E12.5 forebrain: the cerebral microvasculature is tightly embedded into the neuronal tissue of E12.5 wild type embryos (B); the perlecan-deficient vessels are dilated and loosely incorporated in the tissue (C). (D–E) Laminin immunostained BMs of neuroepithelium and blood vessels in ventral forebrain sections. A region with a blood leakage in a mutant embryo is shown (arrow in E). (F–G) Whole-mount picture of wild type (F) and perlecan mutant (G) at E17.5. Hemorrhages (arrow in G) and edema in the skin is evident in the mutant embryo. (H–I) Hematoxilin and eosin staining of the skin sections of E16.5 embryos. The skin of the perlecan-null embryo shows increased interstitial spaces (arrowheads) and dilated vessels (arrow). Bars: (B and C) 125 µm; (D and E) 40 µm; (H and I) 50 µm.

**Figure 2:**
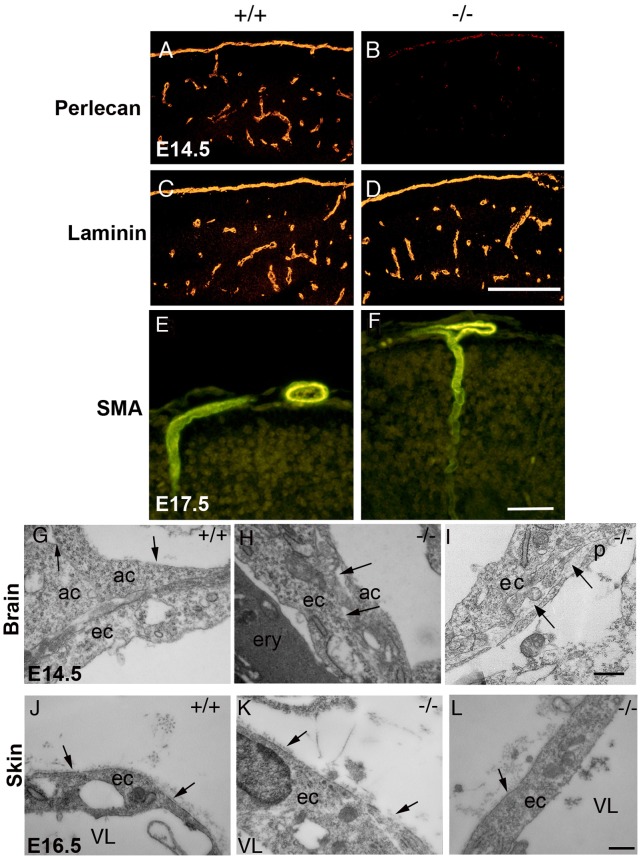
Immunostaining and ultra-structural analysis of microvessels. (A–B) Perlecan is expressed in the sub-endothelial BMs of normal brain capillaries and is absent in the mutant tissue. (C–D) Laminin-1 is present in both wild type and perlecan-null BMs. (E–F) SMA is expressed in the microvessels sprouting into the brain parenchyma in wild type E17.5 (E), as well as mutant brains (F). (G–I) Ultrastructural analysis of endothelial cells from E14.5 brain capillaries. Note the tight association between the endothelial cell (ec) and the directly adjacent cells (ac) in the wild type brain capillary (G). The BM is visible on the upper surface of the adjacent cells (arrows in G). In the mutant brains, gaps are evident between the endothelial cells and the adjacent cells and pericytes (p) (arrows in H and I). (J–L) Electron micrographs of E16.5 skin microvessels. Electron dense material (arrows) is deposited at the abluminar side and appears as a BM-like structure in the wild type (J) as well as in the perlecan-null vessels (K,L).Vessel lumen (VL). Bars: (A–F) 250 µm; (G–L) 400****nm.

At E12.5-13.5, 7 out of 28 perlecan-null embryos (25%) showed distensions in microvessels and small hemorrhages in the brain. Figs. 1C and 1E depict different telencephalic areas with distended microvessel and blood leakage. At E15.5 the vessel distensions in the brain became even more evident (Fig. S2 A–E). We measured the mean perimeter of vessels in 4 areas of 62.5 mm^2^ size per section (fig. S2A,B). The vessel perimeter in the perlecan-null brain was 1.75-fold higher than the wild type littermates (Fig. S2E). At E17.5 39% (5 out of 13) mutants suffered from severe hemorrhages and swollen body (Fig. 1G). Hematoxilin/eosin staining of E16.5 skin sections showed increased interstitial spaces (Fig. 1J, K) and large and irregular blood vessels (see arrow in Fig. 1I). Immunostaining for nidogen in skin sections demarcated the vessel perimeter in the wild type embryos (Fig. S2F). However, in perlecan-null embryos the deposition of nidogen was only visible around the small vessels but not in the distended vessels (see arrow in Fig. S2G). To investigate whether the BM components were normally deposited in perlecan-deficient vessels, the cerebral microvessels were immunostained with antibodies against different BM proteins. Perlecan expression was detected in BMs lining microvessels of E14.5 wild type embryos (Fig. 2A). No staining was observed in perlecan-deficient embryos (Fig. 2B). Laminin-1, collagen IV, nidogen and collagen XVIII were present in the microvasculature of both control and perlecan-null embryos (Fig. 2C–D, and data not shown). In liver, kidney and skeletal muscle we found no evidence of blood vessel distension or abnormal deposition of BMs components (not shown).

Since smooth muscle cells are essential for vascular stability, we investigated the presence of pericytes surrounding the mutant microvessels using smooth muscle α-actin (SMA) immunostainings and electron microscopy. At E17.5, brain vessels of normal and perlecan-null mice stained strongly for SMA, indicating that they were surrounded by pericytes (Fig. 2F). Other markers for pericytes, such as desmin, NG2 and PDGFR1B, confirmed the presence of VSMCs in brain microvessels of E17.5 perlecan-null embryos (not shown). Ultrastructural analysis of 4 normal and 4 perlecan-null E14.5 brain sections also confirmed the presence of pericytes in perlecan-deficient vessels (Fig. 2I). While in normal brain vessels endothelial cells remained tightly associated with adjacent cells, in perlecan-null brains large gaps between endothelial cells and adjacent tissue were visible (Fig. 2 G–I). In skin continuous BMs were present both in wild type and in perlecan-null vessels (Fig. 2 J–L). Moreover, microvessels in choroid plexus and major blood vessels such as the dorsal aorta examined by histology, immunohistochemistry and electron microscopy appeared normal (not shown). These data show that perlecan supports cohesion between endothelial and adjacent cells in some microvessels, while it is dispensable for the recruitment of pericytes or smooth muscle cells.

### Teratomas Derived from Perlecan-null ES Cells Grow Normally and Attract Host Vessels

**Figure 3:**
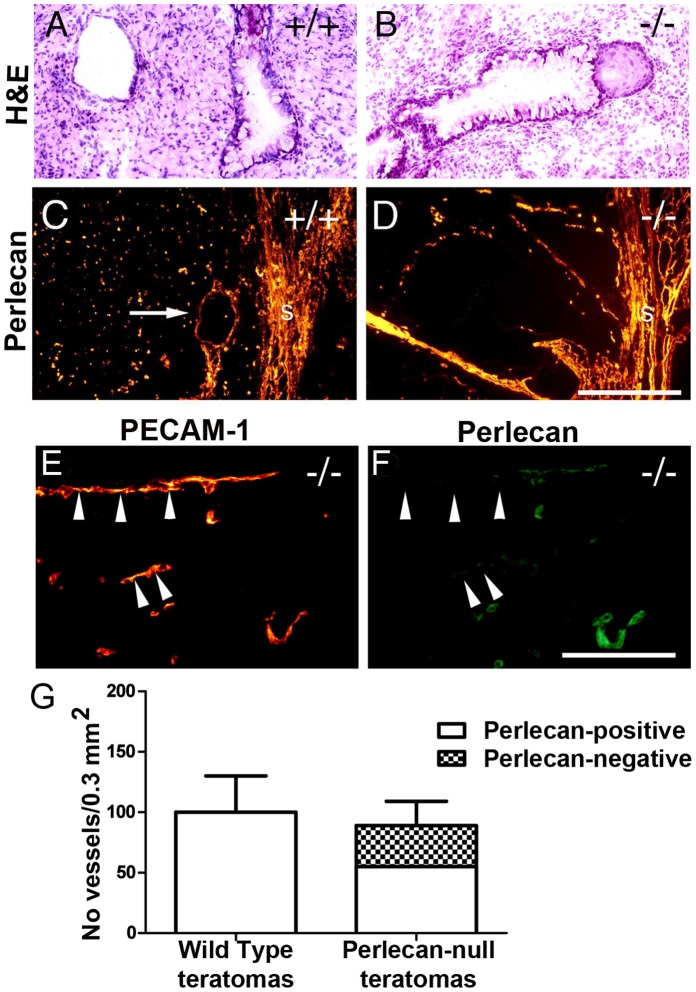
Histological and immunohistochemical analysis of teratomas. Hematoxylin/eosin staining shows teratomas derived from wild type (A) and mutant (B) ES cells that are composed of a variety of differentiated cells. (C–D) Perlecan immunofluorescent labeling in wild type (C) and mutant (D) teratomas. In wild type perlecan is expressed in the stroma (s), and in BMs surrounding vessels (arrow in C) and other structures including glands. Perlecan-null teratomas also have perlecan expression in the tumor stroma (s) and vessels. (E–F) Double fluorescent labeling with PECAM-1 in red (E) and perlecan in green (F) reveal that the vasculature of the perlecan-null tumors is composed of a mixture of perlecan-positive (host-derived) and perlecan-negative (ES cell-derived; arrowheads) endothelial cells. (G) Quantification of perlecan-positive and perlecan-negative vessels per area in wild type (n = 10) and mutant teratomas (n = 10). Bars: (A–D) 250 µm; (E–F) 125 µm.

To investigate whether perlecan influences the development of teratomas, wild type and perlecan-null ES cells were injected subcutaneously into syngeneic male mice. Wild type and perlecan-null tumors had a similar size and weighed 1.3±1.1 g and 1.4±1.0 g (n = 10 of each genotype), respectively. Hematoxylin/eosin staining revealed that wild type and perlecan-null teratomas were composed of a variety of differentiated cells and tissues including glandular structures, neuronal cell nests, muscle cells and areas of connective tissue (Fig. 3A,B). Some (6 out of 14) perlecan-null teratomas appeared looser in structure with more extensive glandular structures compared to wild type tumors.

To analyze the deposition of BM components, sections were stained for perlecan, laminin and collagen IV. In wild type teratomas, perlecan (Fig. 3C) and other BM components (not shown) were present in the stroma, and in the BMs along blood vessels (see arrow in Fig. 3C) as well as in several other structures such as glands. In perlecan-null teratomas, perlecan (Fig. 3D), laminin and collagen IV (not shown) were also present in the stroma and along BMs. The presence of perlecan-positive BMs indicates that it was host derived. To evaluate whether the perlecan-null ES cells also contributed to the tumor vasculature, we double immunostained perlecan-null teratomas with PECAM-1 and perlecan antibodies. As shown in Fig. 3E,F the BM of some PECAM-positive vessels lacked perlecan (see the arrowheads), indicating that both perlecan-null ES cells together with the invading host endothelial cells contributed to the tumor vasculature. The vessels without perlecan were of normal size.

To determine whether the vascular invasion from the host into the tumor tissue differed between wild type and perlecan-deficient teratomas the number of perlecan-positive and negative vessels was counted to distinguish between ES cell-derived tumor tissue (perlecan-negative) and host-derived, stromal tissue (perlecan-positive). The total number of vessels was determined in 10 wild type and 10 mutant teratomas by counting PECAM-positive microvessels in 3 sections of each teratoma. Wild type teratomas contained 100±30/0.3 mm^2^ vessels and perlecan-null teratomas contained 86±20 vessels per 0.3 mm^2^. The vessels in the perlecan-deficient teratomas were distributed into 40% perlecan-negative and 60% perlecan-positive vessels (Fig. 3G). Thus, the contribution of the mutant ES cells to form vessels in the tumor was slightly but not significantly reduced, compared to the wild type ES cells contribution. These data show that perlecan is not essential for teratoma growth, and that perlecan-null endothelial cells participate in blood vessel formation.

### Perlecan Modulates Vascular Formation in Embryoid Bodies

**Figure 4:**
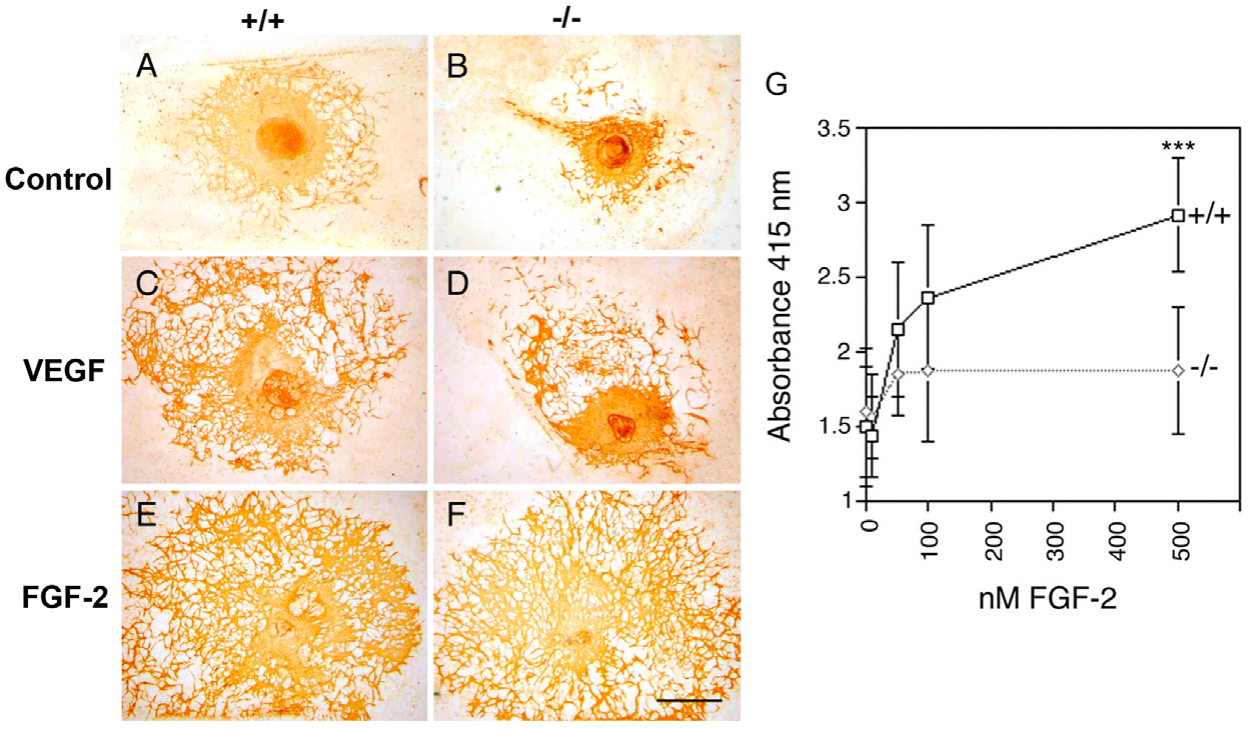
In vitro analysis of vessel formation in embryoid bodies. EBs were generated from control (+/+) and perlecan mutant (−/−) ES cells. (A–F) The data are shown from PECAM-1 immunostainings of 5+12 days EBs cultured in 5% FCS-containing DMEM. A prominent vascular network is formed in wild type EBs (A), while mutants form endothelial networks that are less dense and smaller (B). (C–D) Treatment with 20 ng/ml VEGF_165_ stimulates the formation of large PECAM-positive vascularized areas both in control and mutant EBs. (E–F) Treatment with 20 ng/ml FGF-2 strongly induces the formation of extensive vascular networks in both control and perlecan-deficient EBs. (G) Binding of FGFR1-AP to control (+/+) and perlecan mutant (−/−) 5+9 days EBs. The EBs were grown until confluency in 96-well tissue culture plates and fixed. The fixed EBs were incubated with increasing concentration of FGF-2 and exposed to FGFR1-AP at a concentration of 300 µg/ml. After washing to remove unbound receptor bound FGFR1-AP was measured with AP chromogenic substrate. The data are means of 8 EBs per data-point. Statistical differences were tested by Students t-test (***, p<0.001). The experiment was repeated 3 times. Bars: (A–F) 1 mm.

Vasculo- and angiogenesis is modulated by a large number of growth factors and ECM proteins [35]. They have overlapping functions and therefore, loss of one component may not result in defects. In order to minimize the *in vivo* complexity, we generated normal and perlecan-null EBs to examine endothelial cell differentiation and vessel formation in the presence of different growth factor levels. ES cells were cultured for 5 days in suspension culture to aggregate into EBs and then plated on tissue culture slides and cultured for up to 12 days. Scattered PECAM-positive cells differentiated in cultures of both wild type and perlecan-null ES cells to a similar extent and arranged into a similar vascular network that started to form branches 4 days after plating. Twelve days after plating, a prominent vascular network with extensive branching was evident in wild type and perlecan-deficient EBs (Fig. 4A,B). The size of this network was quantified by counting the number of vessel branch points and the size of the vascularized areas (Table 1). At all serum concentrations (20% to 0.5% FCS-containing DMEM) the number of vessel branches and the PECAM-positive vascularized areas were significantly smaller in the perlecan-null EBs (Fig. 4A,B and Table 1). As shown in the Table 1, the perlecan-deficient EB vascularization was about 60% of that of the wild type. In rescue experiments perlecan with or without HS side chains purified from the Engelbreth-Holm-Swarm sarcoma was added to the EB cultures. Interestingly, neither of the perlecans rescued the vascular plexus development of the null EBs at any concentration (not shown) suggesting that exogenous perlecan was improperly integrated into tissues.

**Table 1:** Number of PECAM-positive vessel branches and size of vascularized areas in wild type and perlecan-null EBs.

Treatment	No of vessel branches/1.5 mm^2^					Vascularized area (mm^2^)				
	+/+	FC	−/−	FC	p^*^	+/+	FC	−/−	FC	p^*^
0.5% serum	154±52		94±37		0.0001	1.5±0.8		0.9±0.6		0.0032
2% serum	162±53		89±45		0.0001	1.9±0.6		1.3±0.6		0.0003
5% serum	170±39		110±40		0.0001	2.6±1.4		1.8±1.4		0.0001
10% serum	320±58		161±50		0.0001	3.5±1.4		2.0±1.0		0.0001
20% serum	324±79		207±39		0.0001	4.2±1.6		2.3±1.2		0.0001
1 nM VEGF_165_ a	218±49	1.3	170±45	1.6	0.0046	4.9±2.7	1.9	2.8±2.5	1.6	0.0001
3 nM VEGF_165_ a	243±76	1.4	150±41	1.4	0.0001	6.1±4.0	2.4	3.6±2.3	2.0	0.0001
0.3 nM FGF2^a^	173±53	1.0	111±24	1.0	0.0001	3.0±1.9	1.2	1.8±1.0	1.0	0.0001
0.6 nM FGF2^a^	156±49	-1.1	105±17	1.0	0.0005	3.4±1.5	1.3	3.6±2.0	2.0	0.8049
1.2 nM FGF2^a^	193±30	1.1	185±25	1.7	0.9053	7.1±2.9	2.7	7.6±2.9	4.2	0.0759
1.2 nM FGF2+1nM VEGF_165_ a	237±46	1.4	227±47	2.1	0.1137	11±3.8	4.1	10±2.9	5.7	0.6457

Data are presented as mean±SD collected from three independent experiments, including a total of 40–60 EBs analyzed per experiment. Statistical differences were evaluated using the two-tailed Mann-Whitney Test, p-values are given (p*).

a All growth factor treatments were performed in 5% serum-containing medium.

FC: Fold change with respect to ES cells growing in 5% serum of the same genotype.

To test whether addition of angiogenic growth factors can rescue the vascularization defect, EBs were cultured in the presence of VEGF_165_ and/or FGF-2 in 5% FCS-containing medium, the numbers of vessel branches and the vascularized PECAM-positive areas were measured and the fold change with respect to EBs growing in 5% FCS of the same genotype was calculated (Fig. 4C–F and Table 1). A 3 nM VEGF_165_ treatment for 12 days increased the number of vessel branches and the size of the PECAM-positive area by 1.4-fold and by 2.4-fold, respectively in wild type EBs, and by 1.4-fold and 2-fold, respectively, in mutant EBs. Although the relative increase compared to control values occurred to a similar extent in normal and mutant EBs, the number of vessel branches and the size of the vascularized plexus in perlecan-deficient EBs did not reach the final values of wild type EBs. Treatment of wild type EBs with 1.2 nM FGF-2 had also a stimulatory effect on vascular plexus formation, as the number of vessel branches and the size of the vascularized area increased 1.1 and 2.7-fold, respectively. In perlecan-null EBs the increase was so massive (1.7 and 4.2-fold, respectively) that the number of vessel branches and the sizes of vascularized areas became similar to those of control EBs. These data suggest that perlecan is modulating FGF-2 but not VEGF_165_ function.

To test whether the heparan sulfate (HS) side chains of perlecan play a significant role for the presentation of FGF-2 to the FGFR1, we added FGF-2 and soluble FGF-receptor1-alkaline phosphatase (FGFR1-AP) fusion protein to the EB cultures. This assay allows quantifying the number of HS chains in EBs cultures that bind FGF-2 and FGFR1-AP by determining the AP activity. Fig. 4G shows that addition of increasing levels of FGF-2 (10–500 nM) to wild type EBs responded with an increasing AP activity. In perlecan-null EBs, however, FGF-2 binding to HS chains was saturated at 50 nM of FGF-2 and no further increase in AP activity was observed (Fig. 4G). These data indicate that the numbers of HS chains capable of mediating the formation of the tri-molecular complex is reduced in perlecan-deficient EBs.

## Discussion

In the present report we have investigated vascular development in mice, teratomas and EBs lacking perlecan expression. We found that perlecan has no critical function during early vascular development in mice or in teratomas. At later stages of embryonic development, when blood vessels have matured and the blood pressure is rising, perlecan-null mice develop dilations and ruptures in some vessels suggesting that perlecan is critical for maintaining microvessel integrity. Finally, in vitro differentiation of perlecan-null ES cells in EBs revealed a significantly reduced efficiency of blood vessel formation which could be rescued by the addition of FGF-2 but not VEGF_165_ suggesting that perlecan modulates angiogenesis by presenting FGF-2 but not VEGF_165_ to endothelial cells.

### Perlecan is not Required for Early Vasculo- and Angiogenesis in Mice

The consequence of a loss of perlecan expression for vascular development was difficult to predict from previously published data. The strong expression of perlecan by migrating endothelial cells and its ability to bind and modulate angiogenic growth factors suggested a crucial role for perlecan already during early vessel formation. Knockdown of the perlecan mRNA in zebrafish showed relatively normal development of axial vessels, dorsal aorta and posterior cardinal vein, but abnormal intersegmental vessel sprouts [28], [36], and the vascular invasion into the growth plate of perlecan-null mouse embryos was also impaired [37]. In the last two cases a role for VEGF signaling was demonstrated to regulate developmental angiogenesis.

In our mouse mutant, however, we found that the entire vasculature was indistinguishable between control and perlecan-null mice at E9.5–10.5. All major vessels including the dorsal aorta and the vascular plexus around the brain developed normally. The mutant mice showed no defects in angiogenesis, sprouting, and remodeling and generated vessels of different sizes. The extra-embryonic circulation was also identical between normal and perlecan-null mice. Several additional heparan sulfate (HS) proteoglycans including agrin and collagen XVIII are expressed by endothelial cells, incorporated into the BM, and are also able to bind and present growth factors. Mice lacking agrin or collagen XVIII are available, and it would be possible in the future to intercross them with our perlecan-deficient mice in order to test whether compensation among these proteoglycans is the reason for the absence of an early vascular phenotype.

### Perlecan is Essential for the Integrity of Mature Blood Vessels

After E12.5, microvessels in brain and skin of perlecan-null mice appeared distended and loosely incorporated into the adjacent tissue. At E15.5 microvessel perimeters in brain were 1.75-fold larger when compared to wild type. Ultrastructural analyses revealed abundant gaps between endothelial and adjacent cells. In E17.5 skin, 39% of perlecan-null embryos showed formation of edemas and interstitial hemorrhages. Interestingly, not the entire vasculature of the mutant embryos was affected. For example, microvessels of choroid plexus and also several other tissues, such as liver, skeletal muscle or kidney, developed neither macroscopic nor ultrastructural defects.

An important step in vascular morphogenesis is the recruitment of pericytes, which, in conjunction with endothelial cells, facilitate tube stabilization. Factors secreted by endothelial cells, such as PDGF-B play a critical role in this event and mice lacking either PDGF-B or PDGF-Rβ have reduced numbers of microvascular smooth muscle cells leading to microaneurysm formation and capillary rupture at late embryonic stages [38], [39]. Perlecan binds PDGF-B with high affinity [40], but immunostaining and electron microscopy revealed the presence of pericytes surrounding microvessels of brain and skin in all perlecan-deficient embryos analyzed.

Since the defects in distinct perlecan-null vessels develop during or shortly after the maturation of microvessels, abnormal BM assembly could cause the defects. Several other mice carrying mutations in BM components and cell adhesion receptors have similar defects in vascular integrity, such as the Fibulin-1-null mutants [41], the deletion of the laminin α4 chain that produces a drastic reduction of type IV collagen and nidogen in vascular BMs [42], mice lacking type XV collagen [43], and absence of αv integrin [44] or β8 integrin [45]. According to our data the distribution of laminin, nidogen, type IV and type XVIII collagen and αv integrins was not altered in the perlecan mutants suggesting that perlecan is not required for vascular BM assembly. However, its presence could maintain vascular integrity. We found evidence supporting this hypothesis using perlecan-deficient ES cells and showed that perlecan is not required for laminin accumulation on the cell surface but rather for appropriate organization of laminin-1 into complex structures [46]. Since vascular BMs have distinct compositions of ECM proteins, it is possible that in the absence of perlecan, some BMs fail to resist hemodynamic pressure and finally degrade whereas others are more independent of perlecan. Other tissues under heavy strain, such as the heart wall suffer from BM instability in the perlecan-null embryos [8].

It is well documented the role of perlecan to inhibit vascular smooth muscle cells (VSMCs) proliferation in a HS-dependent way [47]–[49]. Accordingly, perlecan-deficient embryos show SMC hyperplasia in the heart outflow tract [6] and in the aorta [48], and the perlecan-HS-deficient mice show intimal hyperplasia following carotid artery injury [49], [50]. It is possible that an excess of SMCs during vessel formation could contribute to alter blood vessel stability and to the formation of aneurisms. However, we did not observed accumulation of pericytes in microvessels. In addition control of SMCs proliferation has been shown to depend on HS chains [48], and mice with HS-defective perlecan do not suffer from hemorrhages [50].

### Normal Host-derived Endothelial Cell Recruitment, Growth and Differentiation in Perlecan-null Teratomas

Several laboratories have reported that perlecan can exert a strong influence on tumor angiogenesis and tumor growth [16], [51]. ES cells have the ability to induce benign teratomas when they are grown ectopically in mice and, therefore, represent a suitable system to test endothelial cell differentiation and to test whether perlecan acts as a structural scaffold, influencing teratoma angiogenesis and growth. In contrast to tumors derived from somatic tissue, the vasculature of teratomas is derived from the host as well as from the ES cells [52], [53]. Like the mutant mice, perlecan-null ES cells were able to differentiate into endothelial cells in teratomas. Furthermore, tumor growth was also normal. This finding is not surprising since the 60% of the tumor vasculature was of host origin and thus surrounded by a perlecan-containing BM. However, perlecan-null endothelial cells also contributed to the vessel lining. Despite this finding, the total number of vessels was not different when compared to wild type teratomas suggesting that the postulated angiogenic activities of perlecan are not crucial in this experimental system. It has previously been noted that the growth rate of vessels in many tumor types is very slow compared to physiological angiogenesis [54]. Such a delay in angiogenesis may very effectively compensate defects in vessel maintenance in tumors. It is also possible that the teratoma-derived vasculature is excluded from alterations due to the presence of other HS proteoglycans in BM.

### Perlecan Modulates Vessel Formation in EBs

Differentiation of ES cells in vitro mimics events in early embryogenesis and has been widely used to analyze the maturation of several cell types such as muscle, epithelial, hematopoietic and endothelial cells. ES cells can be differentiated at high or low serum concentrations, in the presence or absence of angiogenic growth factors and are therefore, often used to unravel specific functions that are redundant in complex in vivo system such as embryos and tumors. We also used this in vitro system to investigate differentiation and endothelial tube formation of perlecan mutant ES cells. Our data clearly demonstrate that perlecan-null ES cells can differentiate into PECAM-positive endothelial cells that organize into large networks of vessel-like tubes of various diameters at high (20%) and low (0.5%) concentrations of serum. The networks formed by the mutant cells, however, contained significantly fewer numbers of vessel branches and the area they covered was significantly smaller than in wild type EBs, indicating that tube formation in vitro is less efficient in the absence of perlecan. This defect of the perlecan mutant ES cells was evident at all serum concentrations tested. Addition of perlecan with or without HS side chains purified from the Engelbreth-Holm-Swarm sarcoma did not rescued the vascular plexus development of the null EBs. This could be explained by the fact that the exogenous perlecans did not incorporate into the matrix of the EBs where perlecan normally is very abundant. This result contrasts the normal vasculogenesis observed in perlecan-null embryos. An explanation for this discrepancy could be that certain components in the culture medium may become limiting during in vitro vessel network formation that are available in excess locally in tissues. One main proposed function of perlecan is to modulate growth factor responses both by preserving them from proteolysis and by mediating high affinity binding to the FGF-receptor. We tested two perlecan-binding growth factors, VEGF_165_ and FGF-2, for their effects on endothelial cell tube formation and growth in control and mutant EBs. Although VEGF_165_ stimulated endothelial cell expansion and sprouting in control and mutant EBs, the mutant endothelial cells responded significantly less than the wild type cells at all concentrations tested. Treatment with 1.2 nM FGF-2, however, stimulated and rescued the defective formation of a vessel-like network by the perlecan-null cells. FGF-2 has previously been shown to support the induction and survival of angioblasts in EBs [55], suggesting that FGF-2 can be the limiting factor that causes the reduced vascular network formation in mutant EBs. Although FGF-2 could be limiting for EB vessel branching in our in vitro system, it may not be limiting during development, perhaps due to a moderate rate of growth or due to a higher availability of FGF-2 locally in embryos. Another explanation could be that angiogenesis is not FGF-2-dependent during development, while it may be postnatally. Such situation has been described in perlecan mutant mice lacking the HS side chains [29], [50], in which the embryonic vasculature develops normally but the vessel numbers are diminished in the granulation tissue after wound healing, or after FGF-2-induced corneal neovascularization postnatally.

The relevance of perlecan HS to trap and to present FGF-2 to its receptor is further supported by the experiments using the FGFR1-AP fusion protein as a tool to estimate the proportion of HS chains capable of mediating binding to the FGF-2 and FGFR1 complex. These experiments show that fewer numbers of permissive HS chains were present in the perlecan mutants. The fewer binding sites mediating formation of the tri-molecular receptor complex (HS/FGF-2/FGFR1) present in the EBs may contribute to the reduced vascular formation in the mutants. However, this reduction can be rescued with high amounts of FGF-2. Moreover, during FGF stimulation, the potentially fewer number of FGF-binding sites in the matrix lacking perlecan may have the opposite effect, namely resulting in higher levels of FGFs available for immediate receptor presentation and activation and rescue of the vascular defect. The apparent absence of vascular defects during development in both mutant mouse strains, as well as in the FGF-2 knockout mice [56], highlights the high redundancy operating to ensure the development of vital processes such as vasculo- and angiogenesis.

## Supporting Information

Figure S1 **Vessel formation in perlecan-null embryos.** (A–H) PECAM-1 whole-mount immunostainings of E9.5 control (A, C) and perlecan-null embryos (B, D), and E10.5 and 14.5 control (E, G) and perlecan-null yolk sacs (E, H). C and D shows the capillary plexus of the head. The PECAM-positive vasculature is indistinguishable between wild type and perlecan-null embryos and yolk sacs. (I) Quantification of microvessel density, as number of branch points per mm^2^ in wild type (n = 6) and perlecan-null (n = 6). Statistical differences in number of vessel branch-points between +/+ and −/− were tested by two-tailed Mann-Whitney test. Bars: (A–B) 250 µm; (C–D) 50 µm; (E–H) 25 µm.
(TIF)

Figure S2 **Vessel dilations in perlecan-null embryos.** (A–B) Nissl staining of the pallium shows the areas where diameter of blood vessel was measured in wild type and perlecan-null E15.5 embryos. (C–D) Laminin immunofluorescence shows perineural and blood vessel BMs in wild type (C) and perlecan-null (D) embryos. (E) The perimeter of the neuroepithelial microvessels in the square were measured in three sections (n = 3 for wild type and n = 3 for perlecan-null). Statistical differences between +/+ and −/− were tested by two-tailed Mann-Whitney test (*, *p*<0.05). (F–G) Nidogen immunofluorescent staining showing the BM lining the blood vessels in E16.5 wild type (F) and perlecan-deficient skin (G). The epidermis (e) is at the left-upper corner. Note the deposition of nidogen in the wild type vessels (arrow in F), and its absence in some vessels of the mutant skin (arrow in G). Bars: (A–B) 250 µm; (C–D) 40 µm; (F–G) 20 µm.
(TIF)
