# Supplementary figures and images for: Perlecan Maintains Microvessel Integrity In Vivo and Modulates Their Formation In Vitro
Source: PLoS One. 2013 Jan 8;8(1):e53715. doi: 10.1371/journal.pone.0053715 (PMC3540034; doi:10.1371/journal.pone.0053715)

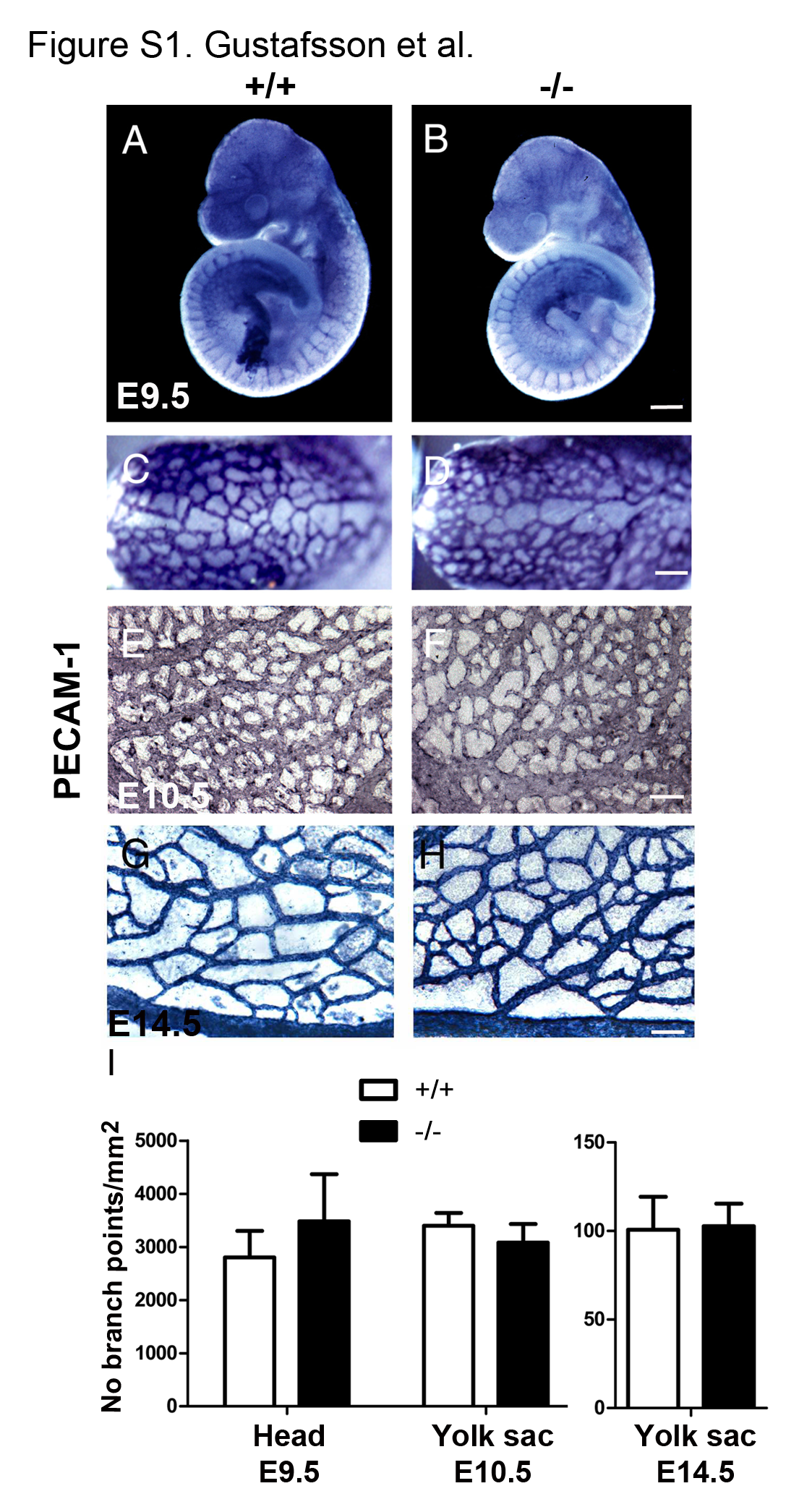

Supplement: Figure S1 — Vessel formation in perlecan-null embryos. (A–H) PECAM-1 whole-mount immunostainings of E9.5 control (A, C) and perlecan-null embryos (B, D), and E10.5 and 14.5 control (E, G) and perlecan-null yolk sacs (E, H). C and D shows the capillary plexus of the head. The PECAM-positive vasculature is indistinguishable between wild type and perlecan-null embryos and yolk sacs. (I) Quantification of microvessel density, as number of branch points per mm2 in wild type (n = 6) and perlecan-null (n = 6). Statistical differences in number of vessel branch-points between +/+ and −/− were tested by two-tailed Mann-Whitney test. Bars: (A–B) 250 µm; (C–D) 50 µm; (E–H) 25 µm. (TIF) [file pone.0053715.s001.tif]

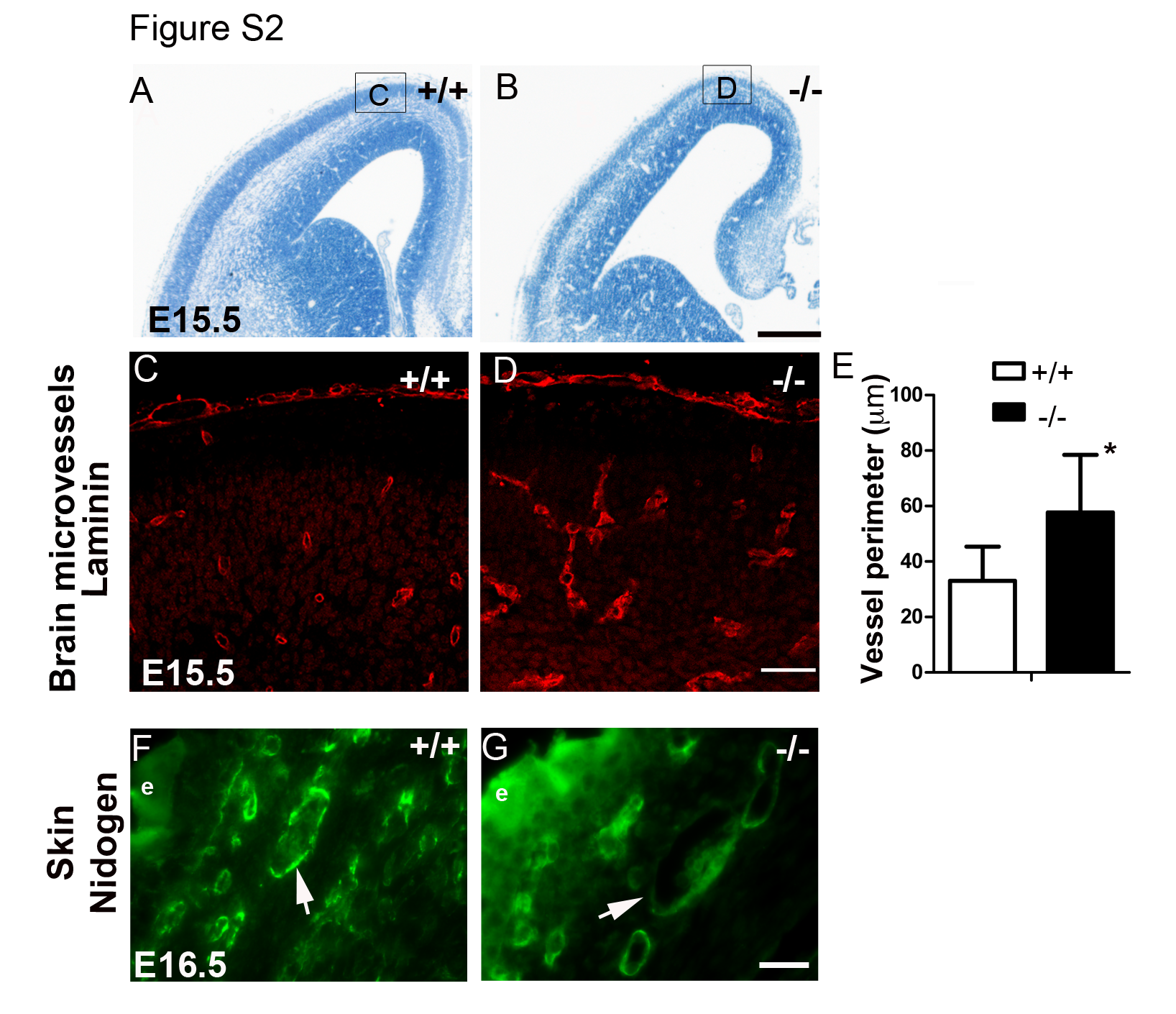

Supplement: Figure S2 — Vessel dilations in perlecan-null embryos. (A–B) Nissl staining of the pallium shows the areas where diameter of blood vessel was measured in wild type and perlecan-null E15.5 embryos. (C–D) Laminin immunofluorescence shows perineural and blood vessel BMs in wild type (C) and perlecan-null (D) embryos. (E) The perimeter of the neuroepithelial microvessels in the square were measured in three sections (n = 3 for wild type and n = 3 for perlecan-null). Statistical differences between +/+ and −/− were tested by two-tailed Mann-Whitney test (*, p<0.05). (F–G) Nidogen immunofluorescent staining showing the BM lining the blood vessels in E16.5 wild type (F) and perlecan-deficient skin (G). The epidermis (e) is at the left-upper corner. Note the deposition of nidogen in the wild type vessels (arrow in F), and its absence in some vessels of the mutant skin (arrow in G). Bars: (A–B) 250 µm; (C–D) 40 µm; (F–G) 20 µm. (TIF) [file pone.0053715.s002.tif]
